# Identification and Characterization of Circular RNAs As a New Class of Putative Biomarkers in Human Blood

**DOI:** 10.1371/journal.pone.0141214

**Published:** 2015-10-20

**Authors:** Sebastian Memczak, Panagiotis Papavasileiou, Oliver Peters, Nikolaus Rajewsky

**Affiliations:** 1 Systems Biology of Gene Regulatory Elements, Berlin Institute for Medical Systems Biology, Max Delbrück Center for Molecular Medicine, Robert Rössle Straße 10, D-13125 Berlin, Germany; 2 Department of Psychiatry, Charité - Universitätsmedizin Berlin, Campus Benjamin Franklin, D-12203 Berlin, Germany; French National Center for Scientific Research - Institut de biologie moléculaire et cellulaire, FRANCE

## Abstract

Covalently closed circular RNA molecules (circRNAs) have recently emerged as a class of RNA isoforms with widespread and tissue specific expression across animals, oftentimes independent of the corresponding linear mRNAs. circRNAs are remarkably stable and sometimes highly expressed molecules. Here, we sequenced RNA in human peripheral whole blood to determine the potential of circRNAs as biomarkers in an easily accessible body fluid. We report the reproducible detection of thousands of circRNAs. Importantly, we observed that hundreds of circRNAs are much higher expressed than corresponding linear mRNAs. Thus, circRNA expression in human blood reveals and quantifies the activity of hundreds of coding genes not accessible by classical mRNA specific assays. Our findings suggest that circRNAs could be used as biomarker molecules in standard clinical blood samples.

## Introduction

Regulatory RNAs such as microRNAs (miRNAs) or long non-coding RNAs (lncRNAs) have been implicated in many biological processes and human diseases such as cancer (reviewed in [1,2]. Recent studies have drawn attention to a new class of RNA that is endogenously expressed as single-stranded, covalently closed circular molecules (circRNA, reviewed in [3]). Most circRNAs are probably products of a ‘back-splice’ reaction that joins a splice donor site with an upstream splice acceptor site [4,5]. Circular RNA is known for several decades from viroids, viruses and tetrahymena [6–8], but until recently only few mammalian circRNAs were reported [9–16]. Sequencing based studies lately revealed that circRNAs are abundantly and prevalently expressed across life, oftentimes in a tissue- and developmental stage-specific manner [17–25]. The vast majority of circRNAs consists of 2–4 exons of protein coding genes, but they can also derive from intronic, non-coding, antisense, 5’ or 3’ untranslated or intergenic genomic regions [20],[26]. Although not fully understood, the biogenesis of many mammalian circRNAs depends on complementary sequences within flanking introns [4],[25],[27–30] and their expression can be modulated by antagonistic or activating trans-acting factors such as ADAR [30] and Quaking [29].

Although the function of animal circRNAs is largely unknown, it was demonstrated that the circRNAs CDR1as (ciRS-7) and SRY can act as antagonists of specific miRNAs by functioning as miRNA sponges [20],[31]. Moreover, stable knockdown of CDR1as caused a migration defect in cell culture [20] and a circRNA produced from the muscleblind transcript can bind muscleblind protein and likely regulate its expression levels [4]. Besides these specific functions for the few in-depth analyzed circRNAs, a recent study uncovered a putatively more general competition mechanism between linear RNA splicing and co-transcriptional circular RNA splicing [4].

Since circularity renders RNA largely resistant to exonucleolytic activities, circRNAs are stable molecules as demonstrated by their long half lives in cells [11],[19],[20]. This led us to ask whether circRNAs could serve as putative biomarker molecules in clinically relevant samples. Here, we report the discovery of thousands of circRNAs in clinical whole blood specimen which were processed following standard procedures. Strikingly, we observe hundreds of cases where circular RNA isoforms are readily detectable but the corresponding linear gene products are virtually absent. Thus, blood circRNA expression may contain disease relevant information which cannot be assessed by canonical RNA analysis.

## Results

### Thousands of circRNAs are reproducibly detected in human peripheral whole blood

**Fig 1:**
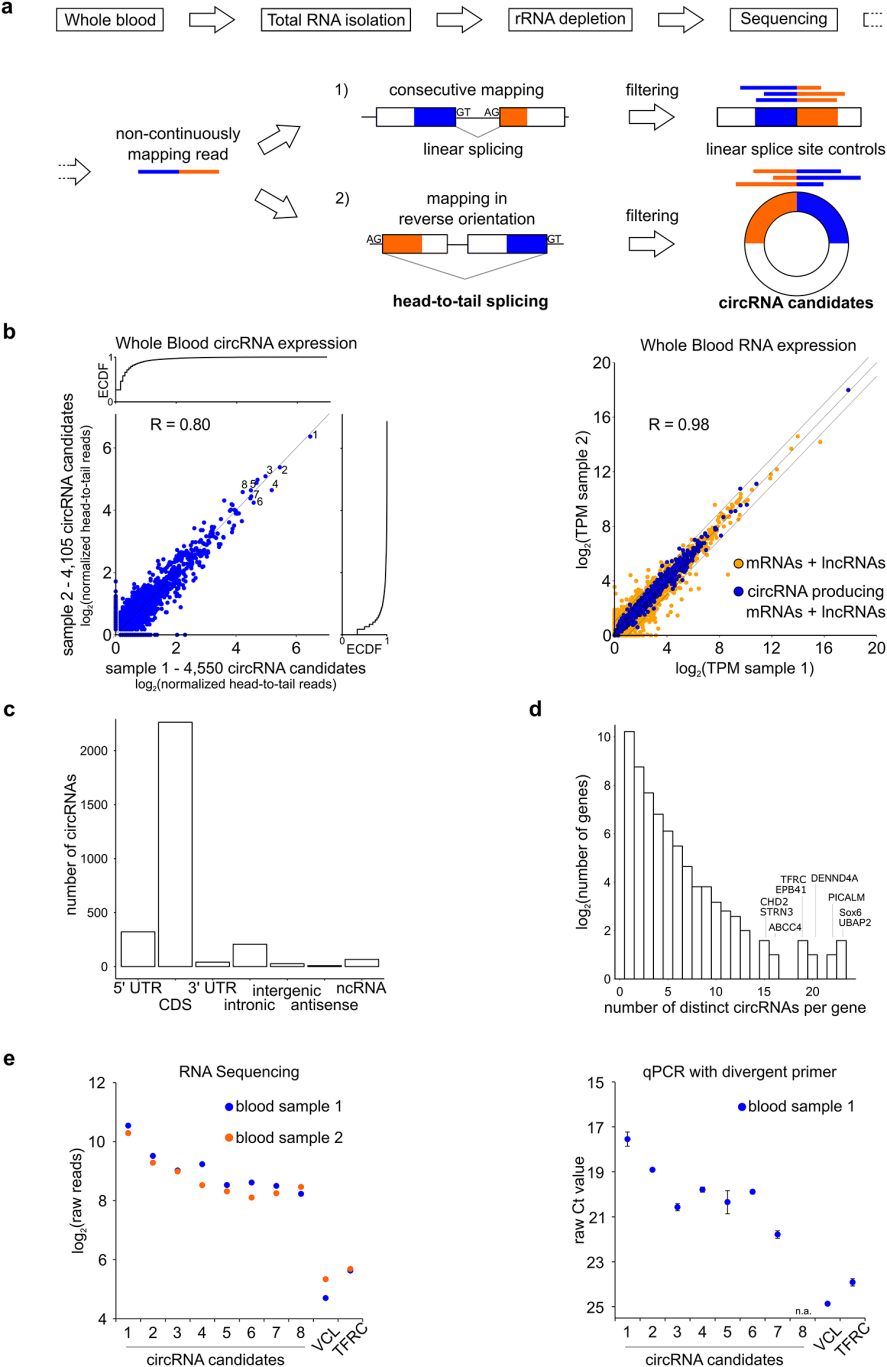
Thousands of circRNAs are reproducibly detected in human blood. (a) Total RNA was extracted from human whole blood samples and rRNA was depleted. cDNA libraries were synthesized using random primer and subjected to sequencing. circRNAs were detected as previously described [20]. Sequencing reads that map continuously to the human reference genome were disregarded. From unmapped reads anchors were extracted and independently mapped. Anchors that align consecutively indicate linear splicing events 1) whereas alignment in reverse orientation indicates head-to-tail splicing as observed for circular RNAs 2). After extensive filtering of linear splicing events and circRNA candidates (Methods) the genomic coordinates and additional information such as read count and annotation are documented (S1 Table) and are available at the circular RNA database circbase.org [38]. (b) circRNA candidate expression in human whole blood samples from two donors, ECDF = empirical cumulative distribution function. circRNA candidates tested in this study are annotated as numbers. Right panel: mRNA and lncRNA (n = 17,282) expression per gene in two blood samples in transcripts per million (TPM), RNAs with putative circular isoforms (n = 2,523) are highlighted in blue; R-values: Spearman correlation for RNAs found in both samples. (c) ENSEMBL genome annotation for reproducibly detected circRNA candidates (see also S1 Fig). Number of circRNAs with at least one splice site in each category is given. (d) Number of distinct circRNA candidates per gene. y-axis = log_2_(circRNA frequency+1). Gene names with the highest numbers are highlighted. (e) Expression level of top 8 circRNA candidates measured with sequencing (left panel) and divergent primer in qPCR (right); Ct = cycle threshold, linear control genes VCL and TFRC were measured with convergent primer.

We first determined whether circRNAs are present in standard clinical blood specimen. To this end, we prepared total RNA from two biologically independent human peripheral whole blood samples and depleted ribosomal RNAs (Methods). The samples were reverse transcribed using random primer to allow for circRNA detection and sequencing libraries were produced (Fig 1a). The raw reads were fed into our *in silico* circRNA detection pipeline [20]. In short, the program filters reads that map continuously to the genome but saves unmapped reads. From those, terminal 20-mer anchors are extracted and independently aligned to the genome. If the anchors map in reverse orientation and can be extended to cover the whole read sequence, they are flagged as head-to-tail junction spanning, *i*.*e*. indicative for circRNAs. Anchors that aligned consecutively were used to determine linear splicing as an internal library quality control and to assess linear RNA isoform expression (Table 1).

**Table 1:** circRNAs are highly expressed in blood.

	number of total reads (millions)	% map to rRNA	#reads over linear splice junctions (millions)	#reads over head-to-tail splice junctions	% head-to-tail of linear splice junction reads	#circRNA candidates
**Blood sample 1**	57.85	11.52	2.37	53,077	**2.19**	4,550
**Blood sample 2**	48.04	6.32	2.08	47,158	**2.22**	4,105
Liver sample 1*	86.12	3.35	5.36	6,186	**0.12**	839
Liver sample 2*	103.53	6.95	10.71	15,148	**0.14**	1,557
Cerebellum sample 1*	87.22	0.49	2.78	62,616	**2.20**	6,792
Cerebellum sample 2*	122.00	0.14	3.01	44,932	**1.47**	5,786

Summary of sequencing results for blood RNA compared to liver and cerebellum samples, for each tissue data from two donors were analyzed (* denotes ENCODE dataset, see S4 Table.)

From the RNA of two human donors we identified 4550 and 4105 unique circRNA candidates, respectively, by at least two independent reads spanning a head-to-tail splice junction (Fig 1b). In both datasets the number of total reads and linear splicing events were similar, indicating reproducible sample preparation (Table 1, S3 Table). When considering RNAs found in both samples, we observed a high correlation of expression for both linear (R = 0.98) as well as circRNAs (R = 0.80, Fig 1b). Between the two samples 1265 circRNAs (55%) with more than 5 reads overlap and 2442 (39%) circRNAs supported by at least 2 unique reads are shared (S1 Table, S1 Fig, technical reproducibility is shown in S2 Fig). The later set will be considered as reproducibly detected circRNAs in the following analysis. circRNA candidates are derived from genes covering the whole dynamic range of RNA expression (Fig 1b, **right panel**). We then compared the blood data to published ENCODE project datasets from cerebellum, representative of neuronal tissues that in general have high circRNA expression [25] and to a non-neuronal primary tissue, liver. Overall we detect a strikingly high circRNA expression in blood compared to liver and cerebellum, measured as percent head-to-tail spanning reads of linear splicing reads (Table 1). We detect >15-fold higher general circRNA expression in blood compared to the liver samples, a level comparable to the circRNA rich cerebellum.

Further, as observed in other human samples, we find that most circRNAs are derived from protein coding exonic regions or 5’ UTR sequences (Fig 1c [20],[25]). GO term enrichment analysis on reproducibly detected, top expressed circRNAs and the same number of top linear RNAs showed significant enrichment of different biological function annotations (S3 Fig). Together with the broad expression spectrum of corresponding host genes, this finding argues that circRNA expression levels are largely independent of linear RNA isoform abundance.

The predicted spliced length of blood circRNAs of 200–800 nt (median = 343 nt) is similar to that in liver or cerebellum (median = 394/448 nt) and previous observations in HEK293 cell cultures and other human samples (S4 Fig and [20]). However, we observed a high number of circRNAs per gene, with 23 genes giving rise to more than 10 circRNAs (‘circRNA hotspots’, Fig 1d).

To assess the reproducibility of the sequencing results we designed divergent circRNA specific primer and measured relative abundances of the top eight expressed circRNAs compared to linear control genes in qPCR (Fig 1e). circRNA candidate 8 could not be unambiguously amplified from cDNA, most likely due to overlapping RNA isoforms and was therefore excluded from further analysis. For the remaining seven circRNA candidates, we tested circularity using previously established assays: 1) resistance to the 3’-5’ exonuclease RNase R and 2) Sanger sequencing of PCR amplicons to confirm the sequence of predicted head-to-tail splice junctions. With these assays we validated 7/7 tested candidates suggesting that the overall false positive rate in our data sets is low (S5 Fig). Interestingly, these circRNAs are expressed from gene loci that so far were not shown to have a specific blood related function (S2 Table) but show expression levels that by far exceed expression of housekeeping genes such as VCL or TFRC (4-100-fold, Fig 1e).

### Circular-to-linear RNA expression is high in blood

**Fig 2:**
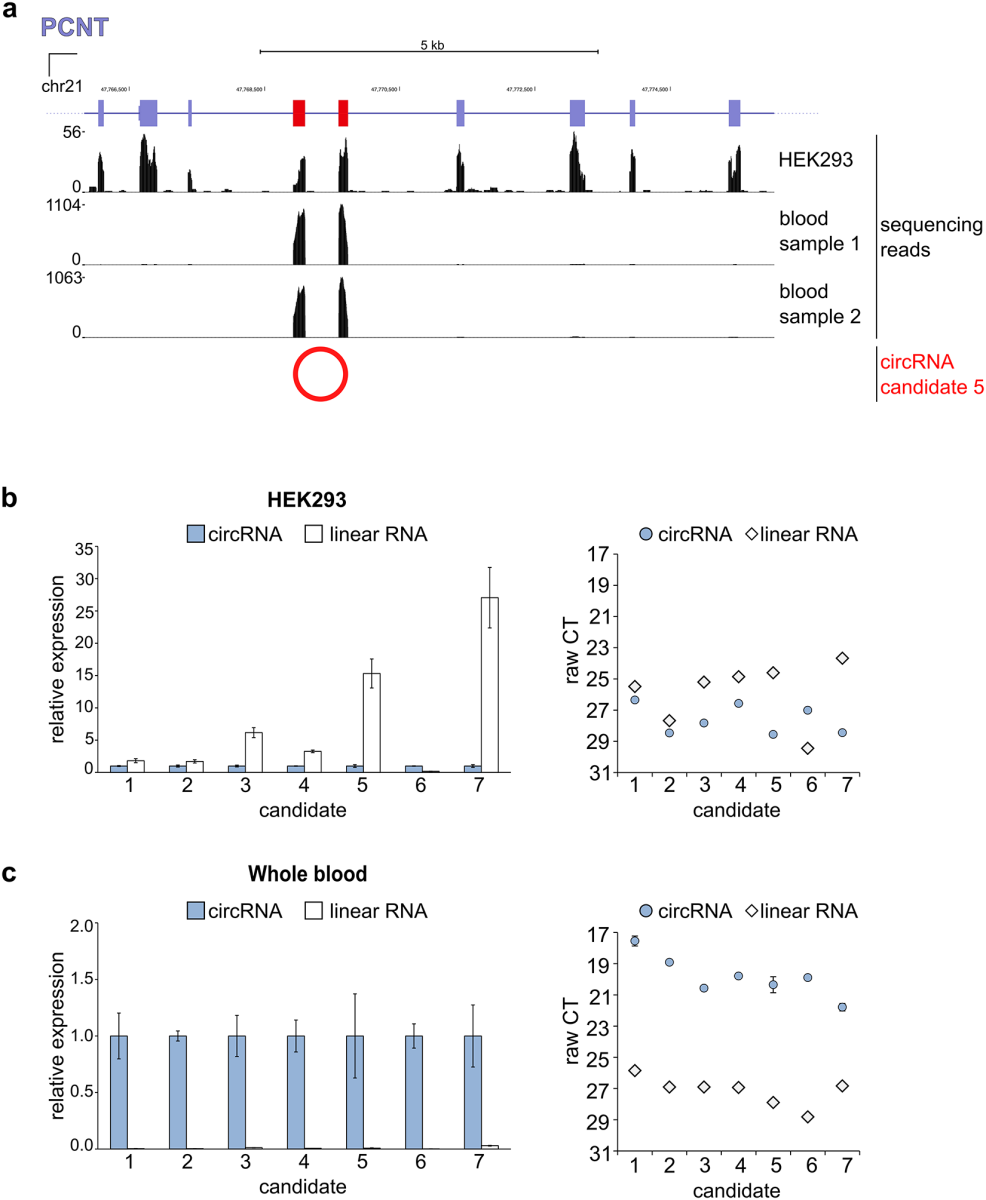
Top expressed blood circRNAs dominate over linear RNA isoforms. (a) Example for the read coverage of a top expressed blood circRNA produced from the PCNT gene locus (http://genome.ucsc.edu/ [36]). Data are shown for the human HEK293 cell line [30] and two biologically independent blood RNA preparations. (b) Relative expression and raw Ct values of top expressed blood circRNAs and corresponding linear isoforms in HEK293 cells and whole blood (c).

When inspecting the read coverage in blood sequencing data, we noticed that oftentimes the expression of circularized exons was outstandingly high compared to the coverage of neighboring exons expressed in linear RNA isoforms of the same gene. For example, we observed that the two exons of circRNA candidate 5, which is product of the PCNT locus were densely covered with sequencing reads in the blood samples, while the upstream and downstream exons were barely detected (Fig 2a). This particular expression pattern was not observed in HEK293 cells, where all exons were equally covered. We investigated this observation further by qPCR, comparing linear to circular RNA expression with isoform specific primer sets in HEK293 and whole blood samples (Fig 2b and 2c). With this independent assay we confirmed the dominant expression of the tested candidates which was found to be at least 30-fold higher than the cognate linear isoforms. In contrast, this circRNA domination was not found in HEK293 cells where the same RNAs were probed, which argues for a tissue-specific pattern. Approx. 30% of blood circRNAs are also found in cerebellum while this fraction was around 10% for liver with higher fractions for both cases when constraining the analysis to highly expressed blood circRNAs (S6a–S6d Fig,comparison between total RNAs in S7 Fig). In summary, circRNAs found in human whole blood in part overlap circRNAs expressed in cerebellum or liver, but also contain hundreds of other circRNAs.

**Fig 3:**
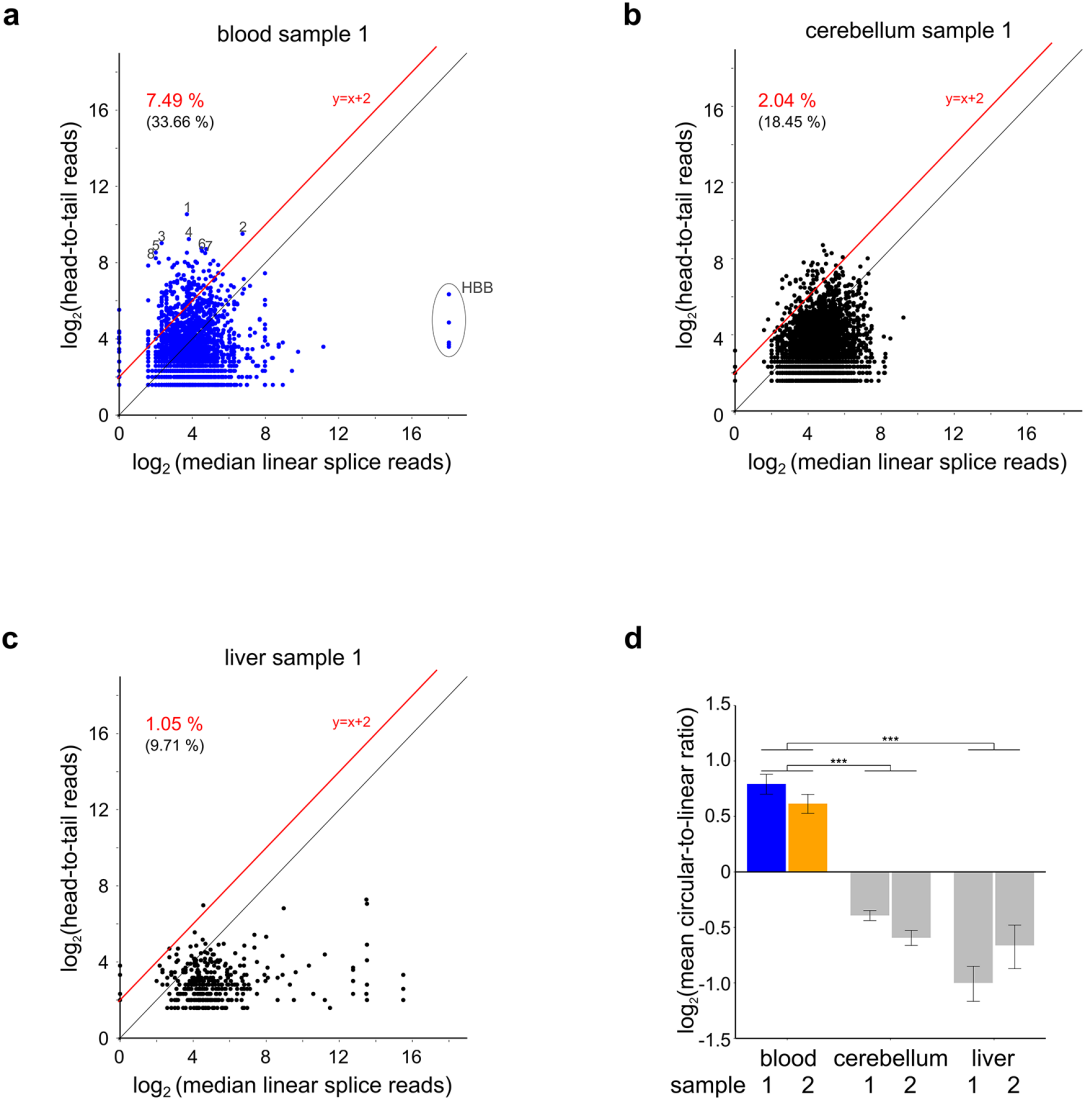
Circular to linear RNA isoform expression is high in blood compared to other tissues. (a) Comparison of circular to linear RNA isoforms in blood. circRNAs were measured by head-to-tail spanning reads. As a proxy for linear RNA expression median linear splice site spanning reads were counted. Data are shown for one replicate each of blood, cerebellum (b) and liver (c). Relative fraction of circRNA candidates with higher expression than linear isoforms are given as insets (>4x in red, >1x in black in brackets). In (a) eight tested circRNA candidates are indicated by numbers, and circRNAs derived from hemoglobin are marked. (d) mean circular-to-linear RNA expression ratio for the same samples, in two biological independent replicates. Error bars indicate the standard error of the mean, *** denotes P <0.001 permutation test on pooled replicate data (Methods). For clarity, panels (a-c) represent expression datasets for one replicate per sample (Table 1).

We next analyzed the relative circular to linear RNA isoform abundance on a transcriptome wide scale. To this end, we compared read counts that span head-to-tail junctions and are therefore indicative of circRNAs, to the median number of read counts on linear splice site junctions on the same gene, the latter serving as a proxy for linear RNA expression (Methods). We observed that many blood circRNAs are highly expressed while corresponding linear RNAs show average or low abundances (Fig 3a), a finding that was recapitulated by qPCR assays validating our approach (Fig 2c, S8 Fig). For the control samples cerebellum and liver this pattern was not observed (Fig 3b and 3c) as revealed by comparing the mean circular-to-linear RNA ratio, which we found to be significantly higher in blood than in the tested control tissues (Fig 3d). In summary, we observed that blood has an outstanding general tendency to express circRNAs at high levels while the corresponding linear transcripts are much more lowly expressed. This tendency was only found (to a much lower extent) in cerebellum but not in liver RNA as well as RNA from many other tissues or cell lines that we have analyzed.

Our results show that circRNAs are reproducibly and easily detected in clinical standard blood samples and therefore suggest that they may have the potential to serve as a new class of biomarker for human disease.

## Discussion

Recent publications show that circRNAs can be detected in plasma and saliva samples [32,33]. However, in both specimens only few (less than 100) circular RNAs with canonical splice sites were reported, which dramatically limits any further analysis. The circular transcriptome of whole blood presented here, suggests that the search for putative circRNA biomarker in peripheral blood is much more suitable to yield informative results. Using RNA-Seq of clinical standard samples we reproducibly found around 2400 circRNA candidates expressed in human whole blood and moreover observed, that the overall circRNA expression level in blood is unexpectedly similar to that of neuronal tissues, where circRNAs are highly abundant [25]. To further assess the reproducibility of the sequencing results we repeated our analysis pipeline on three more, biologically independent samples and found that the high blood circRNA expression is reproducibly observed (total *n = 5*, S4 Table). It will be interesting to determine the origin of blood circRNAs. Accumulating evidence suggests that circRNAs are specifically expressed in a developmental stage- and tissue-specific manner, rather than being merely byproducts of splicing reactions [20,25]. Previously analyzed circRNA from neutrophils, B-cells and hematopoietic stem cells suggest that many circRNAs are constituents of hematocytes [18]. However, there is also the intriguing possibility of circRNA excretion into the extracellular space, e.g. by vesicles such as exosomes which is supported by a recent study [34]. Likewise, aberrant circRNA expression in disease may reflect, either a condition-specific transcriptome change in blood cells themselves, or a direct consequence of active or passive release of circRNA from diseased tissue. Here, we provide the first data to foster future studies aiming to elucidate these scenarios.

Further, we demonstrated that many circRNAs have a high expression compared to linear RNA isoforms from the same locus, a feature that distinguishes blood circRNAs from other primary tissues such as cerebellum or liver. Considering that this was observed for hundreds of blood circRNA candidates (Fig 3a, S1 Table) and that we further restricted our experimental setup to standard samples and preparation procedures, we want to caution that this feature of blood circRNA may distort RNA data analysis. Gene products that are dominated by circRNAs which typically comprise 2–4 exons (example in Fig 2, S9 Fig) will also dominate signals for the specific gene of interest in array assays, Northern Blots or qPCR experiments if the circularized exon expression is measured. Assays designed such that inadvertently circular isoforms are targeted will lead to misinterpretation of the results. A detailed assessment of this phenomenon will be published elsewhere. Further, it is presently not known if the high circular-to-linear RNA ratio in blood reflects a tissue specific RNA population or is an artifact of sample preparation procedures.

Nevertheless, especially given the urgent need for non-invasive biomarker detection for many disease states, we think these findings encourage future in-depth follow up analysis of circRNAs. It will be interesting to search for circRNA biomarkers not only in blood but also in other clinical samples such as cerebrospinal fluid. Although in principle blood circRNA expression might be specifically altered in a plethora of human diseases, investigations of neurological conditions would be of particular interest, since circRNA expression is exceptionally high in neuronal tissues [25] and the circRNA CDR1as was found to have Alzheimer’s Disease specific expression [35].

## Materials and Methods

### Whole blood sample collection

Blood sampling, processing and analysis performed in this study was approved by the Charité ethics committee, registration number EA4/078/14 and all participants gave written informed consent. 5 mL blood were drawn from subjects by venipuncture and collected in K_2_EDTA coated Vacutainer (BD, #368841) and stored on ice until used for RNA preparation. For downstream RNA analysis by sequencing or qPCR assays presented here, 100 μL blood (> 1 μg total RNA) is sufficient.

### Cell Culture

Human HEK293 Flp-In T-REx 293 (Life Technologies, Waltham, Massachusetts) were cultured in Dulbecco’s modified Eagle medium GlutaMax (Gibco) with 4.5 g/l glucose, supplemented with 10% FCS, at 5% CO_2_ and 37°C.

### RNA isolation and RNase R treatment

Total RNA was isolated from fresh whole blood samples. Blood was diluted 1:3 in PBS and 250 μL of the dilution were used for RNA preparation using 750 μL Trizol LS reagent (Thermo Scientific, Waltham, Massachusetts). Samples were homogenized by gentle vortexing and 200 μL chloroform was added. After centrifugation at 4°C, 15 min at full speed in a table top centrifuge, the aqueous phase was collected to a new tube (typically 400 μL). RNA was precipitated by adding an equal volume of cold isopropanol and incubation for ≥ 1 hour at -80°C. RNA pellets were recovered by spinning at 4°C, 30 min at full speed in a table top centrifuge. RNA pellets were washed with 1 mL 80% EtOH and subsequently air dried at room temperature for 5 min. The RNA was resuspended in 20 μL RNase-free water and treated with DNase I (Promega, Fitchburg, Wisconsin) for 15 min at 37°C with subsequent heat inactivation for 10 min at 65°C. HEK293 total RNA was prepared using 1 mL Trizol on cell pellets. For sequencing experiments the RNA preparations were additionally subjected to two rounds of ribosomal RNA depletion using a RiboMinus Kit (Life Technologies K1550-02 and A15020). Total RNA integrity and rRNA depletion were monitored using a Bioanalyzer 2001 (Agilent Technologies, Santa Clara, California). For qPCR analysis the samples were treated with RNase R (Epicentre, San Diego, California) for 15 min at 37°C at a concentration of 3 U/μg RNA. After treatment 5% *C*. *elegans* total RNA was spiked-in followed by phenol-chloroform extraction of the RNA mixture. For controls the RNA was mock treated without the enzyme.

### cDNA library preparation for Deep Sequencing

cDNA libraries were generated according to the Illumina TruSeq protocol (Illumina, San Diego, USA). Sample RNA was fragmented, adaptor ligated, amplified and sequenced on an Illumina HiSeq2000 in 1x 100 cycle runs. Sequencing data have been deposited at GEO under accession number GSE73570.

### Quantitative PCR (qPCR)

Total RNA was reverse transcribed using Maxima reverse transcriptase (Thermo Scientific) according to the manufacturer's protocol. qPCR reactions were performed using Maxima SYBR Green/Rox (Thermo Scientific) on a StepOne Plus System (Applied Biosystems). Primer sequences are available in the S5 Table. RNase R assays were normalized to *C*. *elegans* RNA spike-in RNA. Error bars denote standard deviations (*n* = 3).

### Sanger Sequencing

PCR products were size separated by agarose gel electrophoresis, amplicons were extracted from gels and Sanger sequenced by standard methods (Eurofins, Luxembourg, Luxembourg).

### Detection and annotation of circRNAs

The detection of circular RNA was based on a previously published method [20] with the following details. Human reference genome hg19 (Feb 2009, GRCh37) was downloaded from the UCSC genome browser [36] and was used for all subsequent analysis. bowtie2 (version 2.1.0[37] was employed for mapping of RNA sequencing reads. Reads were mapped to ribosomal RNA sequence data downloaded from the UCSC genome browser. Reads that do not map to rRNA were extracted for further processing. In a second step, all reads that mapped to the genome by aligning the whole read without any trimming (end-to-end mode) were neglected. Reads not mapping continuously to the genome were used for circRNA candidate detection. From those 20 nucleotide terminal sequences (anchors) were extracted and re-aligned independently to the genome. The anchor alignments were then extended until the full read sequence was covered. Consecutively aligning anchors indicate linear splicing events whereas alignment in reverse orientation indicates head-to-tail splicing as observed in circRNAs (Fig 1a **)**. The resulting splicing events were filtered using the following criteria 1) GT/AG signal flanking the splice sites 2) unambiguous breakpoint detection 3) maximum of two mismatches when extending the anchor alignments 4) breakpoint no more than two nucleotides inside the alignment of the anchors 5) at least two independent reads supporting the head-to-tail splice junction 6) a minimum difference of 35 in the bowtie2 alignment score between the first and the second best alignment of each anchor 7) no more than 100 kilobases distance between the two splice sites.

### circRNA annotation

Genomic coordinates of circRNA candidates were intersected with published gene models (ENSEMBL, release 75 containing 22,827 protein coding genes, 7484 lncRNAs and 3411 miRNAs). circRNAs were annotated and exon-intron structure predicted as previously described [20]. Known introns in circRNAs were assumed to be spliced out. Each circRNA was counted to a gene structure category if it overlaps fully or partially with the respective ENSEMBL feature (Fig 1c, S1 Table).

### Published RNA data sets

In this study we used rRNA depleted RNA-seq data from whole blood samples (own data), fetal cerebellum (ENCODE accession: ENCSR000AEW) fetal liver (ENCODE accession: ENCSR000AFB) and HEK293 [30] (S4 Table). Expression values, coordinates and other details of the circRNAs reported here and all associated scripts are available at www.circbase.org/ [38].

### Quantification of circRNA and host gene expression

The number of reads that span a particular head-to-tail junction were used as a measure for circRNA expression. To allow comparison of expression between samples, raw read counts were normalized to sequencing depth by dividing by the number of reads that map to protein coding gene regions and multiply by 1,000,000 (Fig 1b **left**, S2, S6a and S6c Figs). To estimate host gene expression, RNA-seq data were first mapped to the reference genome with STAR [39]. htseq-count [40] was employed to count hits on genomic features of ENSEMBL gene models. The measure transcripts per million (TPM) was calculated for each transcript and sample in order to compare total host gene expression between samples (Fig 1b, **right**).

Circular-to-linear ratios were calculated for each circRNA by dividing raw head-to-tail read counts by the median number of reads that span linear spliced junctions of the respective host gene. For both measures one pseudo count was added to avoid division by zero. circRNAs from host genes without annotated splice junctions according to the ENSEMBL gene annotation, were not considered in this analysis.

For analysis in Fig 3d a permutation test with 1000 Monte-Carlo replications was performed on pooled biological replicate data to approximate the exact conditional distribution. To adjust for different dataset sizes the respective larger data set of each comparison was randomly subsampled. The test was repeated for 1000 subsamples.

## Supporting Information

S1 Fig Reproducibility of circRNA candidate detection. The overlap of 2,442 circRNAs found with at least 2 read counts in both samples is considered as reproducibly detected circRNA set.
(PDF)

S2 Fig Technical reproducibility of circRNA candidate detection. A library of blood Sample 1 was sequenced twice (see S4 Table).
(PDF)

S3 Fig GO annotation of circRNAs and linear RNAs in blood. Significantly enriched GO terms (p<0.05) for circRNAs found in both samples (n = 2,442) and for the same number of top expressed linear RNAs.
(PDF)

S4 Fig Predicted circRNA length. Predicted spliced circRNA length distributions for circRNA candidates detected in liver, cerebellum and blood.
(PDF)

S5 Fig circRNA candidate validation. (a) Top circRNA candidate expression was measured in qPCR using divergent primer on mock or RNase R treated total RNA preparation. 7/8 were successfully amplified while candidate 8 did not yield specific PCR products and is therefore excluded from further analysis. Linear RNAs and previously described circRNAs are shown as controls. (b) PCR amplicons for divergent and convergent primer sets (c—circular, l–linear) of the tested candidates, end point analysis after 40 cycles. (c) Standard curves for tested candidates, div—divergent primer for circular isoforms, con—convergent primer for linear RNA isoforms. (d) PCR amplicons were subjected to Sanger sequencing and checked for the presence of a head-to-tail junction, representative example result is shown.
(PDF)

S6 Fig Comparison of circRNA candidates in blood to liver and cerebellum. (a) Comparison of circular RNA candidates detected in blood (Sample 1) and cerebellum shown for the whole expression range. (b) fraction of circRNA candidates that overlap between the two samples binned by blood expression level. (c, d) Analysis as before but for liver circRNA candidates.
(PDF)

S7 Fig Correlation of linear RNAs in cerebellum and blood and liver and blood. Number of detected transcripts: blood = 29,908; cerebellum = 38,192; liver = 27,880; TPM = transcripts per million.
(PDF)

S8 Fig Comparison circular-to-linear expression by RNA-Seq and qPCR. Raw Ct values (Cycle threshold) and median linear splice junction spanning read counts are given for the respective RNA isoform.
(PDF)

S9 Fig Number of exons per circRNA in blood. Histogram of number of exons per circRNA. Reproducibly detected set (n = 2,442) without intergenic circRNAs (n = 27); median exon number: 2, mean exon number: 2.8.
(PDF)

S1 Table List of circRNAs detected in human blood. Genomic location, ENSEMBL gene identifier, gene symbols and gene biotype are given together with raw read counts for each circRNA candidate in each sample. In a second sheet head-to-tail junction reads and median linear splice site junction spanning reads are given for blood cerebellum and liver samples.
(XLS)

S2 Table Details on top expressed circRNA candidates. (XLS)

S3 Table Reads continuously mapping to hemoglobin genes. (XLS)

S4 Table Summary of RNA-sequencing results. (XLS)

S5 Table List of oligos used in this study. (XLS)
