# Supplementary figures and images for: Identification and Characterization of Circular RNAs As a New Class of Putative Biomarkers in Human Blood
Source: PLoS One. 2015 Oct 20;10(10):e0141214. doi: 10.1371/journal.pone.0141214 (PMC4617279; doi:10.1371/journal.pone.0141214)

unique read count  $\geq 2$

sample 1

sample 2

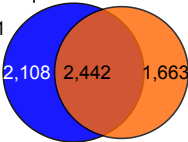

unique read count  $\geq 5$

sample 1

sample 2

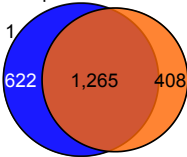

Supplement: S1 Fig — The overlap of 2,442 circRNAs found with at least 2 read counts in both samples is considered as reproducibly detected circRNA set. (PDF) [file pone.0141214.s001.pdf]

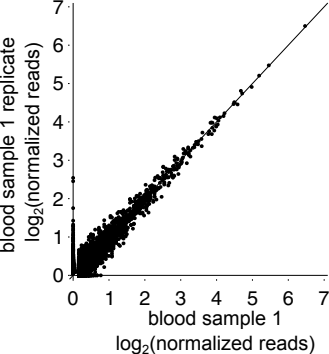

Supplement: S2 Fig — A library of blood Sample 1 was sequenced twice (see S4 Table). (PDF) [file pone.0141214.s002.pdf]

**Top expressed circRNAs**

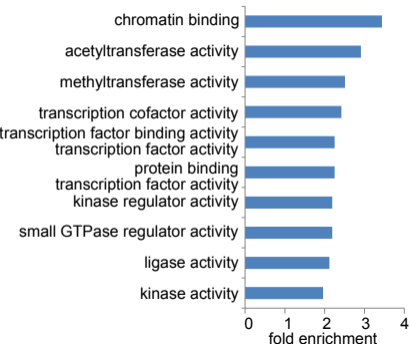

**Top expressed linear RNAs**

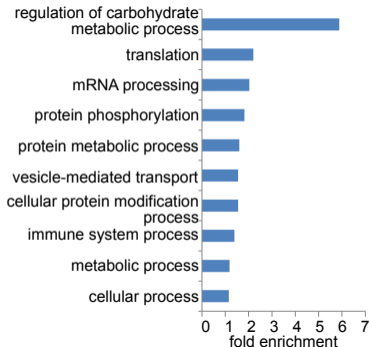

Supplement: S3 Fig — Significantly enriched GO terms (p<0.05) for circRNAs found in both samples (n = 2,442) and for the same number of top expressed linear RNAs. (PDF) [file pone.0141214.s003.pdf]

blood circRNA

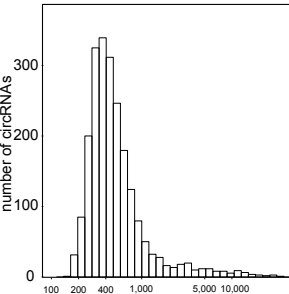

cerebellum circRNA

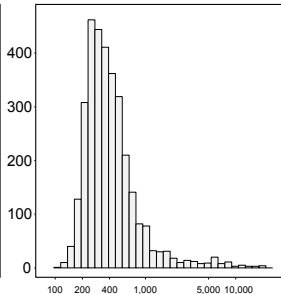

liver circRNA

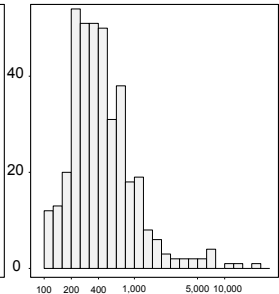

Supplement: S4 Fig — Predicted spliced circRNA length distributions for circRNA candidates detected in liver, cerebellum and blood. (PDF) [file pone.0141214.s004.pdf]

**a**

cerebellum (6,792 circRNA candidates)

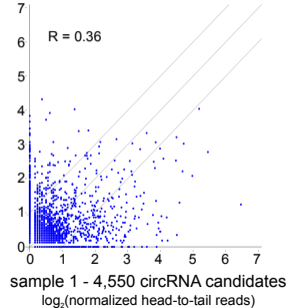**b**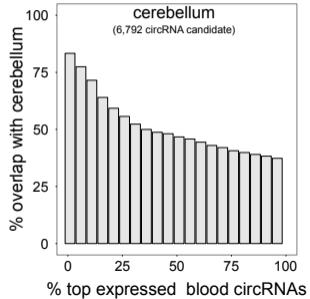**c**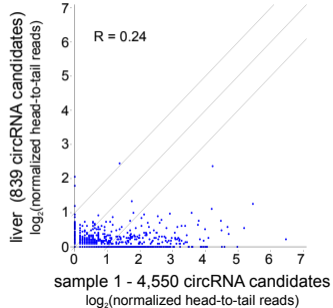**d**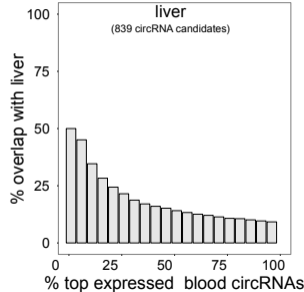

Supplement: S6 Fig — (a) Comparison of circular RNA candidates detected in blood (Sample 1) and cerebellum shown for the whole expression range. (b) fraction of circRNA candidates that overlap between the two samples binned by blood expression level. (c, d) Analysis as before but for liver circRNA candidates. (PDF) [file pone.0141214.s006.pdf]

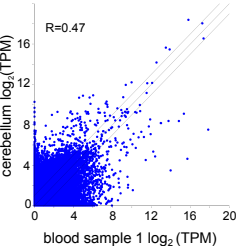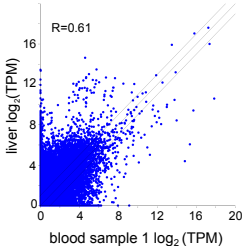

Supplement: S7 Fig — Number of detected transcripts: blood = 29,908; cerebellum = 38,192; liver = 27,880; TPM = transcripts per million. (PDF) [file pone.0141214.s007.pdf]

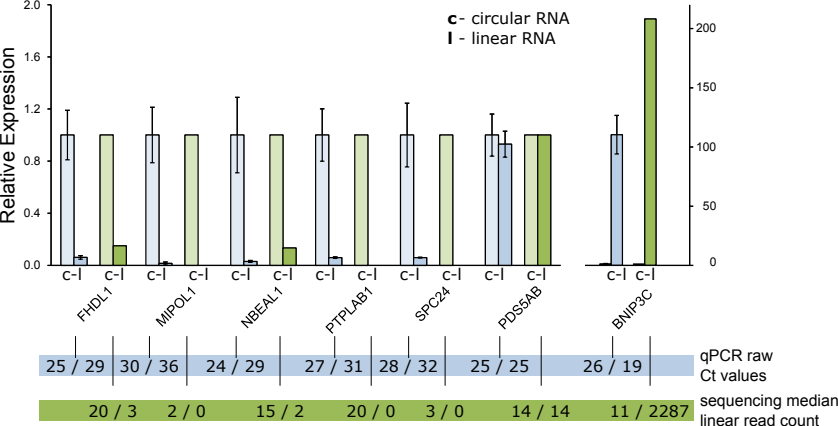

Supplement: S8 Fig — Raw Ct values (Cycle threshold) and median linear splice junction spanning read counts are given for the respective RNA isoform. (PDF) [file pone.0141214.s008.pdf]

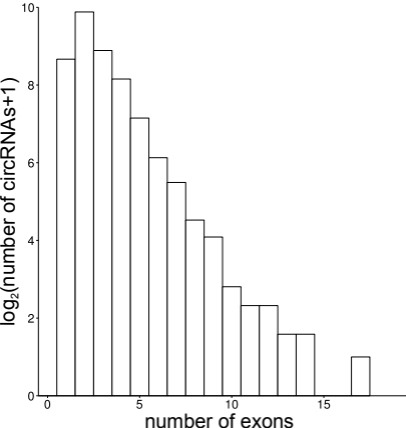

Supplement: S9 Fig — Histogram of number of exons per circRNA. Reproducibly detected set (n = 2,442) without intergenic circRNAs (n = 27); median exon number: 2, mean exon number: 2.8. (PDF) [file pone.0141214.s009.pdf]
