# Supplementary material for: Identification and Characterization of Circular RNAs As a New Class of Putative Biomarkers in Human Blood
Source: PLoS One. 2015 Oct 20;10(10):e0141214. doi: 10.1371/journal.pone.0141214 (PMC4617279; doi:10.1371/journal.pone.0141214)

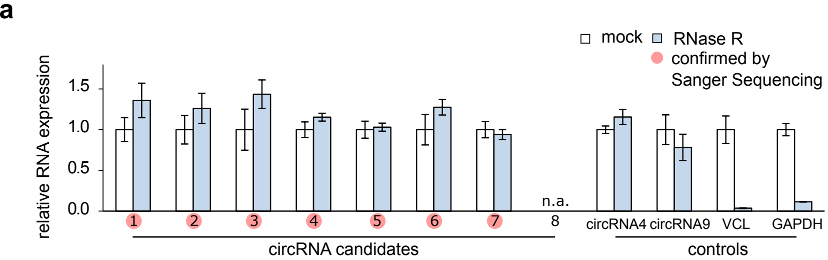

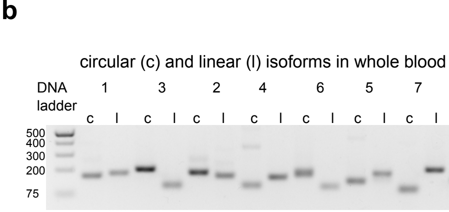

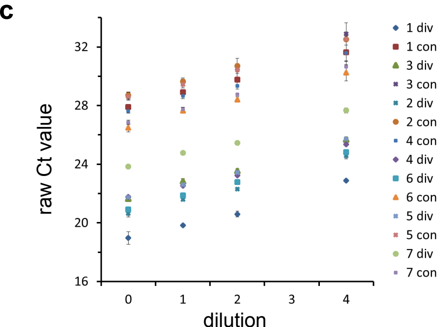

	R^2	slope
1 div	0.99	0.98
1 con	1.00	0.93
2 div	0.99	0.97
2 con	1.00	0.96
3 div	1.00	0.99
3 con	1.00	1.06
4 div	0.99	0.90
4 con	0.99	0.98
5 div	0.99	1.00
5 con	1.00	0.99
6 div	1.00	0.98
6 con	1.00	0.92
7 div	0.99	0.95
7 con	1.00	0.95

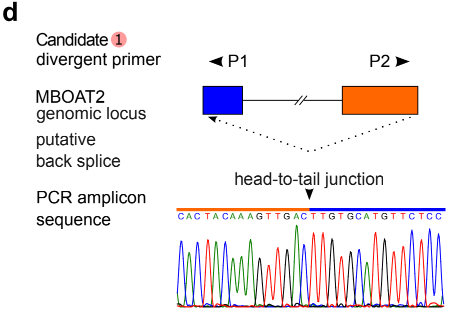

Supplement: S5 Fig — (a) Top circRNA candidate expression was measured in qPCR using divergent primer on mock or RNase R treated total RNA preparation. 7/8 were successfully amplified while candidate 8 did not yield specific PCR products and is therefore excluded from further analysis. Linear RNAs and previously described circRNAs are shown as controls. (b) PCR amplicons for divergent and convergent primer sets (c—circular, l–linear) of the tested candidates, end point analysis after 40 cycles. (c) Standard curves for tested candidates, div—divergent primer for circular isoforms, con—convergent primer for linear RNA isoforms. (d) PCR amplicons were subjected to Sanger sequencing and checked for the presence of a head-to-tail junction, representative example result is shown. (PDF) [file pone.0141214.s005.pdf]
